# Growth and martensitic transformation of ferromagnetic Co-Cr-Ga-Si epitaxial films

**DOI:** 10.1080/14686996.2023.2251368

**Published:** 2023-09-11

**Authors:** Yuru Ge, Klara Lünser, Fabian Ganss, Peter Gaal, Lukas Fink, Sebastian Fähler

**Affiliations:** a Helmholtz-Zentrum Dresden-Rossendorf, Institute of Ion Beam Physics and Materials Research, Dresden, Germany; b Leibniz IFW Dresden, Institute for Metallic Materials, Dresden, Germany; c Faculty of Mechanical Science and Engineering, TU Dresden, Dresden, Germany; d Faculty of Natural Sciences, TU Chemnitz, Chemnitz, Germany; e Leibniz-Institut für Kristallzüchtung (IKZ), Berlin, Germany; f TXproducts UG, Hamburg, Germany

**Keywords:** Co-Cr-Ga-Si, martensitic transformation, reentrant martensite, epitaxial film, ferromagnetism

## Abstract

During cooling, conventional martensitic transformation can only be realized from austenite to martensite. Recently, a so-called reentrant martensitic transformation attracted much interest due to an additional transformation from martensite to austenite during further cooling. Obviously, materials with this reentrant transformation will increase the number of physical effects and possible applications. However, until now, only bulk samples have been available, which are not suitable for applications in micro-devices. In this work, we focus on the Co-Cr-Ga-Si system and examine the suitability of this system for the growth of thin films. We observed that the films grow epitaxially on MgO (100) substrates and exhibit a martensitic transformation if deposited at a sufficiently high temperature or with an additional heat treatment. Films within the austenite state are ferromagnetic while films within the martensitic state just exhibit a very low ferromagnetic order.

## IMPACT STATEMENT

We sincerely make a statement that our work is arranged in the scientific way with highly novelty. So far, the research and reports on reentrant martensitic materials are very less. We make a starting in thin films to arouse enthusiasm for research on this novel shape memory materials. Although we could not observe a reentrant martensitic transformation in the additional experiments due to the limited conditions, we consider our work as a substantial advance in science since we present for the first time a route to prepare epitaxial films from this emerging material, which allowed analyzing its functional properties. So, we think our work is at the high novelty. We consider our manuscript relevant for the broad interdisciplinary readership of Science and Technology of Advanced Materials. First, it is relevant for material scientists, as it addresses reentrant martensite as an emerging class of functional materials. Second, it is interesting for physicist, as our epitaxial films enable probing the underlying mechanism driving a reentrant transition. Third, it is interesting for engineers, as reentrant films are required for microsystems based on this multifunctional material. We thank you for your consideration and your time. We hope that our work is now acceptable for publication in Science and Technology of Advanced Materials.

## GRAPHICAL ABSTRACT

**Figure:**
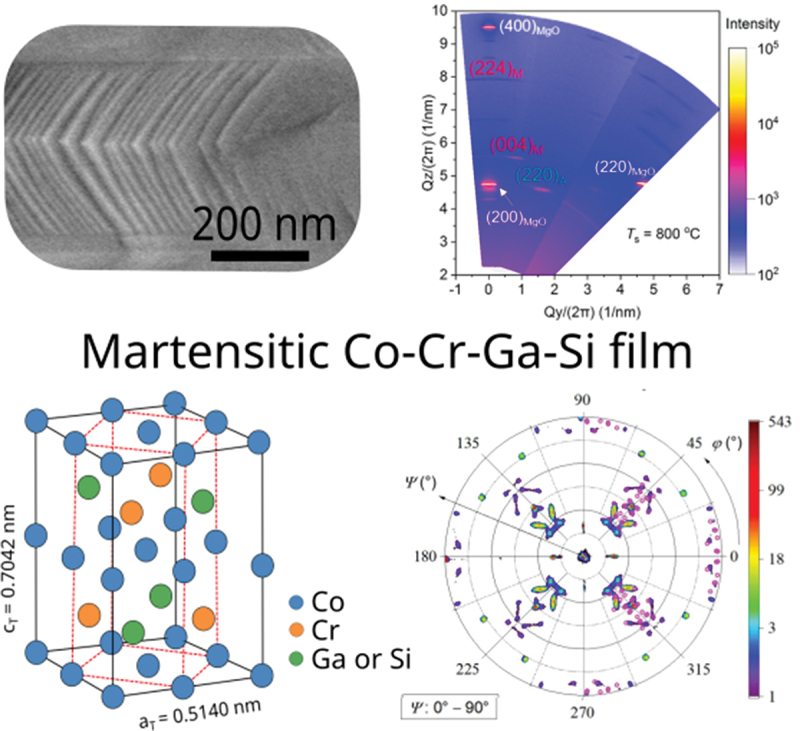

## 1. Introduction

Ferromagnetic and ferroelastic materials are two classes of functional materials which enable many new applications. The combination of both ferromagnetic and ferroelastic behaviors in one material leads to ferromagnetic shape memory effects [1] and results in additional possibilities, including large stroke actuation by a magnetically induced variant reorientation (MIR) [2,3], magnetically induced austenite (MIA) [4] and magnetically induced martensite (MIM) [5]. Recently, the multitude of effects and applications was enriched by the discovery of a so-called reentrant martensitic transformation (RMT) [6–11]. An RMT occurs at temperatures well below the conventional martensitic transformation (MT) and transforms a martensite (M) to austenite (A) by cooling, in contrast to a conventional MT, which can only achieve this by heating. Proposed applications have not been fully explored yet. But there are clearly technological advantages for actuation by both heating and cooling [7], and for elastocaloric applications by obtaining the stiff → soft → stiff sequence of mechanical responses in a single heating or cooling run instead of in a thermal cycle [12]. The materials currently known to exhibit RMT mainly are Co-based alloys [6–11]. Most Co-based alloys can achieve conventional MT. RMT was only observed in the off-stoichiometric Co_2_Cr(GaSi) and Co_2_Cr(AlSi) Heusler alloys by Xu et al [6–11]. However, only bulk alloys are currently available, which limits the applications for micro-processing and flexible devices. Therefore, the aim of this work is to open up the exploration of thin films with RMT.

In this work, we choose off-stoichiometric Co_2_Cr(GaSi) [7] as a model system to explore transformation behaviors and magnetic properties in thin films. We grow the films by DC magnetron sputter deposition with the initial goal of achieving epitaxial growth. As an epitaxial film has a well-defined crystallographic orientation relation with the substrate, it is a model system to study the martensitic microstructure. Epitaxial films also have no large-scale grain boundaries, which is beneficial for variants classification and multi-length-scale analysis [13,14]. For this, we choose MgO (100) as the substrate to obtain a (100)-oriented film, as it has been widely established for the growth of many Heusler alloys, like Co-Mn-Si [15,16] and Ni-Mn-Ga [2,17]. Films are prepared at high temperatures within the austenitic state and accordingly it is worth clarifying that the term ‘epitaxial growth’ refers to this state. When films transform to the martensite state during cooling, different variant orientations form, which are sheared and rotated with respect to the austenite. Consequently, the martensite is not anymore a single crystal in the strict sense. This, however, is not a peculiarity of epitaxial films, but a general feature of single crystals undergoing martensitic transformations. To study the effect of growth parameters on the transformation behavior, we vary the deposition temperature (*T*_s_) between 400°C and 800°C. Rapid thermal annealing (RTA) is used as an additional heat treatment by adjusting the annealing temperature (*T*_a_) as well as the duration (*t*_a_) to see whether it is beneficial for the MT. To probe the MT as well as the magnetic transition, we monitor magnetization in dependence of measurement temperatures (*T*_m_) from 50 to 900 K. We observe that, both for deposition and post-annealing temperatures, up to 700°C, films are ferromagnetic and the compositions remain stable. If above, composition changes and a sharp loss of magnetization is observed. The twin-boundary microstructure, the X-ray diffraction (XRD) peaks and the pole figure patterns synergistically clarify the MT. However, there is no RMT observed during cooling from 300 K to 90 K. From this comprehensive analysis, we deduce the deposition and annealing conditions which enable epitaxial growth, martensitic and magnetic transitions.

## 2. Materials and methods

MgO (100) single-crystals with polished surface of 1 × 1 cm^2^ made by Crystec GmbH are used as substrates. Co-Cr-Ga-Si films are grown by DC magnetron sputter deposition (Bestec GmbH, Germany) in an ultra-high-vacuum chamber with a base pressure of 6 × 10^‑^^9^ mbar. The sputtering power is 70 W. The deposition pressure is 8 × 10^‑^^3^ mbar. The gas within the chamber is Ar with 5 vol % H_2_, which is added to limit metal oxide formation by reduction. During deposition, the substrate holder rotates continuously to achieve a uniform thickness and composition. The target is made from Co, Cr, Ga and Si with a purity better than 99.99%. The nominal target composition is Co_52_Cr_26_Ga_11_Si_11_. The nominal film thickness is 200 nm. For deposition, the substrates are heated to *T*_s_ between 400°C and 800°C. Furthermore, post-annealing is examined on the film of *T*_s_ = 450°C by RTA (CreaTec Fischer & Co. RTA-1000C-H-4Z, Germany) with a vacuum level of ~ 10^‑^^9^ mbar. The composition is measured by energy dispersive X-ray spectroscopy (EDX) and the microstructure is characterized by a scanning electron microscope (SEM) (Zeiss Sigma 300, Germany) with an in-lens detector. The crystal structure is characterized by θ/2θ scans of XRD (Bruker D8 Advance, Germany) in Bragg – Brentano geometry with *χ* tilts from 0° to 10°. The X-rays originate from the Co Kα line with the corresponding wavelength of 0.179 nm. The sample spins during the measurement. To further corroborate the observation from θ/2θ scans, room-temperature out-of-plane reciprocal space mapping (RSM) with a *χ* range from −5° to 45° is done by a SmartLab diffractometer (Rigaku, Japan) with a 2D detector and Cu Kα-radiation. The purpose of tilting *χ* and rotating *ϕ* in θ/2θ scans and of covering *χ* from −5° to 45° in RSM is to capture martensitic variants that are sheared slightly out-of-plane by a MT [13]. To further explore if structural transformations occur away from room temperature, temperature-dependent RSMs were measured by another Rigaku SmartLab diffractometer with Cu Kα-radiation and an Anton Paar DHS 1100 heating stage. The setup allowed for measurements both at low temperatures down to 90 K and at elevated temperatures up to 773 K. The sample was mounted in a graphite dome under vacuum. For these measurements we choose a coplanar diffraction geometry. The pole figure measurements were performed at a four-circle Philips X’pert diffractometer (Malvern Panalytical, UK) with Cu Kα-radiation is used. In order to make a comparison between the measured pole figures and the theoretical orientations of the martensitic variants, we use the nonlinear elasticity-based continuum mechanics as described by Bhattacharya [18] and implemented this theory in a MATLAB code adapted from our previous work [19]. To probe Curie temperature (*T*_c_) and MT temperature (*T*_M_), temperature-dependent magnetic measurements are performed using a vibrating sample magnetometer (VSM) inserted in a physical property measurement system (PPMS; Quantum Design, USA). We used two VSM inserts operated in the standard mode from 50 to 400 K and the oven mode from 300 to 900 K. In both cases, to ensure the film is initially in an austenitic state, it is first heated up to the maximum temperature with the rate of 12 K/min. Then, the film is cooled from maximum temperature to minimum then heated back to maximum with the cooling/heating rate of 2 K/min. During the measurement, a constant external magnetic field of 0.1 T or 2 T is applied in the film plane along one substrate edge.

## 3. Tailoring film properties by varying *T*_s_

We deposited Co-Cr-Ga-Si films on MgO (100) to understand the influence of *T*_s_ on crystal structure, orientation, microstructure and transformation behavior. *T*_s_ varies between 400°C and 800°C. To keep this paper concise, we only show results of the films deposited at 400°C, 600°C and 800°C. XRD θ/2θ scans and SEM results of all the films can be found in the supplementary information.

### 3.1. Crystallographic orientations

**Figure 1.**
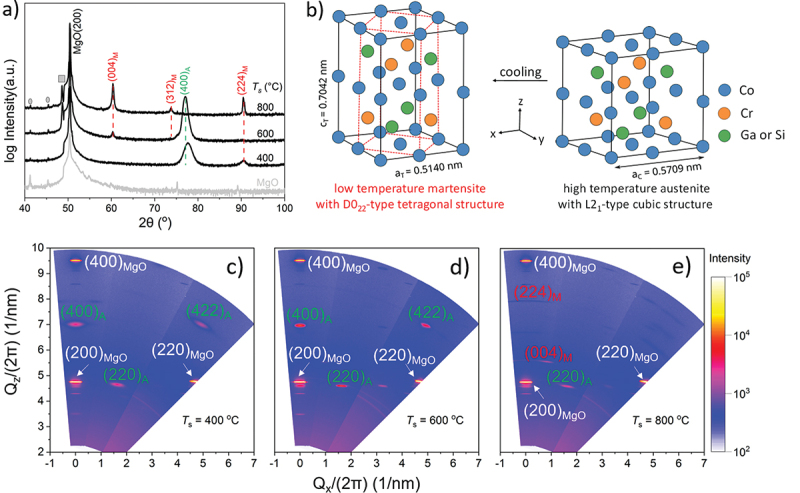
a) Summed-up θ/2θ scans reveal that films deposited at 400°C and 600°C mainly exhibit a single austenitic (400)_A_ reflection, whereas the film grown at 800°C exhibits several martensitic reflections. The reflections marked with grey dots are from the sample holder. The y-axes of all the XRD plots in this paper are in logarithmic scale. b) Schematic of the crystal structure changing when MT occurs. The lattice constant of austenite cell is calculated from θ/2θ scan as a_C_ = 0.5709 nm. For the martensite, the tetragonal cell (black lines on the left) with lattice constants a_T_ = 0.5140 nm and c_T_ = 0.7042 nm is chosen as the cell for martensite and not the D0_22_ unit cell, which is sketched with the red-dashed lines. c–e) RSM measured at room temperature shows that the austenite’s coherence length increases at 600°C compared to 400°C.

In order to study the effect of *T*_s_ on the crystal structure, room-temperature XRD θ/2θ scan is performed. As a MT results in a shear and rotation of variants, a standard measurement in Bragg-Brentano geometry is not suitable for martensitic films. To capture also inclined martensitic variants, we use the approach of Buschbeck et al. [13] of summing up θ/2θ scans with χ tilts from 0º to 10º. Accordingly, in Figure 1(a), also reflections from planes, which are not parallel to the substrate, are observed. Figure 1(a) compares the Co-Cr-Ga-Si films grown at *T*_s_ = 400, 600 and 800°C. The (400)_A_ peak marked with green-dashed line and the (004)_M_, (312)_M_ and (224)_M_ peaks marked with red-dashed lines match the peak positions expected from the literature [7]. They are from the L2_1_-type cubic Co-Cr-Ga-Si austenite and the D0_22_-type tetragonal Co-Cr-Ga-Si martensite, respectively. In Figure 1(a), for the film of *T*_s_ = 400°C, the austenitic peak (400)_A_ is clearly visible and accompanied by a weak (224)_M_ peak. If *T*_s_ = 600°C, we observe the (400)_A_ peak as well as a tiny amount of (004)_M_. This demonstrates that these two samples are mainly austenitic. In addition, as the (400)_A_ peak from the austenite is dominant with only tiny peaks from other orientations, it gives the first hint that films grow epitaxial, or at least with one dominant orientation. If *T*_s_ = 800°C, the (400)_A_ reflection is not seen, but (004)_M_, (312)_M_ and (224)_M_ are observed, revealing that this film is fully martensitic at room temperature. At this point, the presence of a martensitic phase indicates the occurrence of MT above room temperature. There are some adjacent maxima near the MgO (200). A comparison to the diffraction of the pure MgO substrate shows that the broad basis is a part of the substrate reflection. There is one un-indexed peak marked with a square. This peak is neither from Kβ line and W Lα lines nor from other phases of MgO. It also does not belong to L2_1_-type cubic Co-Cr-Ga-Si austenite and D0_22_-type tetragonal Co-Cr-Ga-Si martensite. It might be from another unknown martensitic phase or from other alloy through the phase separation. To preliminarily summarize our results, films deposited below 600°C are mainly austenitic at room temperature. Films deposited at 800°C are martensitic. If *T*_s_ is in between, austenite and martensite coexist. This result is further confirmed by the additional XRD θ/2θ scans of samples with various deposition temperatures between 400°C and 800°C in Figure S1 in the supplementary information. The reflections marked with grey dot appearing in all curves originate from the sample holder (see reference measurement of a pure MgO substrate in Figure 1(a)). Based on the θ/2θ results, a lattice constant of a_C_ = (0.571 ± 0.006) nm (C at subscript refers to cubic austenite) is obtained which is nearly equal to the bulk value of 0.5706 nm [7]. In Figure 1(b), crystal structures of the austenite (right) and the martensite (left) are sketched. It illustrates that during cooling, the cubic austenite cell is elongated along the c-axis. Since the cell volume hardly changes during MT, the cell is simultaneously compressed along the a-axis. The directly elongated tetragonal cell is chosen as the martensite cell to calculate the lattice constants. They are calculated as a_T_ = (0.514 ± 0.009) nm and c_T_ = (0.704 ± 0.012) nm (T at subscript refers to tetragonal martensite). The c_T_/a_T_ ratio is 1.370, which is larger than the bulk value of 1.258 [7]. Throughout the paper, we use this martensitic cell with these lattice parameters, as it intuitively demonstrates the lattice correspondence between austenite and martensite. In addition, the D0_22_-type unit cell of martensite is pointed out, as shown with the red-dashed lines inside the tetragonal cell. Room-temperature RSM in Figure 1(c–e) show that the (400)_A_ and (422)_A_ peaks are not present if *T*_s_ = 800°C, but the (004)_M_ and (224)_M_ peaks are. This confirms the results of the θ/2θ scans that in this case a MT occurs during cooling from room temperature. In addition, regarding the RSM reflex of the (400)_A_ (similarly also for (422)_A_), the reflex shape of the film deposited at 600°C (Figure 1(d)) is thinner and sharper comparing to the film at 400°C (Figure 1(c)). The narrowing in the vertical direction means the austenite’s coherence length increases at 600°C compared to 400°C. The narrowing in the horizontal direction means the mosaicity decreases due to a lower misorientation at 600°C compared to 400°C. This observation is expected by the higher mobility of atoms occurring at increased deposition temperatures. Furthermore, the (220)_A_ reflection is observed in all RSM images. In contrast to (400)_A_ and (422)_A_ peaks, which disappear in martensite film, this is unexpected. The explanation will be given in the following pole figure discussion.

**Figure 2.**
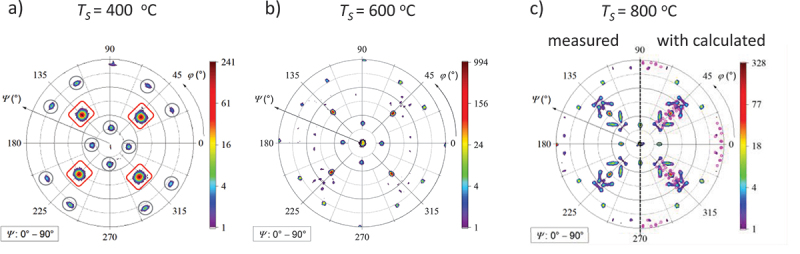
Influence of *T*_s_ on epitaxial growth and orientation relationships: a) the four peaks of the film deposited at 400°C marked with red boxes in the (220)_A_ pole figure originate from epitaxial growth with the orientation relationship MgO (100) [001] || Co-Cr-Ga-Si (100)_A_ [011]_A_. The peak intensities marked with grey circles result from growth twinning [20]. b) at *T*_s_ = 600°C, the appearance of martensitic variants confirms a mixture of austenite and martensite. c) at *T*_s_ = 800°C, the film is mostly martensitic. The comparison of the calculated positions with c/a = 1.258 [7] (pink on the right half) and the measured data (all the other patterns) confirms the martensitic phase. Figure edges are parallel to the substrate edges (MgO [001] and MgO [010]).

To understand the orientation relationship between the substrate and the austenitic film, as well as to identify the phases that are present in the three aforementioned films in more detail, (220)_A_ pole figure measurements are shown in Figure 2. For the film grown at 400°C, in Figure 2(a), the four distinct peaks at *φ* = 45º, 135º, 225º and 315º marked with red boxes originate from the austenite. It clarifies that with respect to the single crystal MgO (100) substrate, the austenitic Co-Cr-Ga-Si cubic cell is rotated by 45º around MgO [100]. In this orientation, the lattice misfit between film and substrate is 4.27%. The film grows epitaxially with the orientation relationship between the substrate and austenitic film of MgO (100) [001] || Co-Cr-Ga-Si (100)_A_ [011]_A_. The weak patterns marked with grey circle originate from the twinning at {211}_A_ planes along < 111> shear directions, which can be attributed to the growth-twinning of the body-centered cubic (bcc) austenite [20,21]. This type of twinning occurs during the deformation of bcc crystals, indicating that it might originate from the misfit, as described above. This is also the reason that in RSM data in Figure 1(c–e), the (220)_A_ is observed in all images. The pole figure confirms that the film is austenitic at room temperature and mostly epitaxial. In Figure 2(b), for comparison, the pole figure of the film deposited at 600°C reveals that the peaks of austenite become sharper and accordingly increase in intensity. We attribute this to a faster diffusion and therefore less small-angle grain boundaries in the film deposited at 600°C compared to 400°C. In addition, we observe the presence of martensitic variants together with the austenite. This demonstrates that the film grows austenitic and then partly transforms into martensite during cooling. As a result, both phases are obtained. The pole figure of the film grown at *T*_s_ = 800°C in Figure 2(c) exhibits a flower-like distribution of intensities, which confirms that during a MT, the austenite splits up into many martensitic variants with different orientations. This is complementary to the corresponding θ/2θ scans data in Figure 1(a), which reveals the presence of the strong (004)_M_, (312)_M_ and (224)_M_ and no (400)_A_. To further confirm that the reflections in the pole figures originate from martensite, we calculate the theoretical values by using nonlinear elasticity-based continuum mechanics [18] with a MATLAB code [19]. For the calculation, c_T_/a_T_ = 1.258 from a literature is used [7]. The result is shown as the pink patterns on the right half of Figure 2(c). The measured patterns are in high agreement with the pink theoretical positions. This confirms that the martensite here exhibits a tetragonal structure. There are still some deviations between experimental and theoretical positions, which results from the differences between c_T_/a_T_ ratio in the present and previous studies. In summary, pole figure results confirm the epitaxial growth in the austenitic state and the variant splitting in the martensitic state.

### 3.2. Microstructures

**Figure 3.**
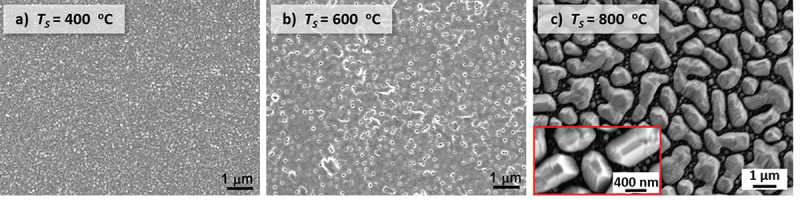
With the increase of *T*_s_ from a) 400°C, to b) 600°C to c) 800°C, the microstructure changes from a granular topography to a continuous film with some gaps and holes, and then finally to the isolated islands due to dewetting; c) additionally shows the martensitic variants connected by twin boundaries. The red box in c) is a zoom-in image to clarify the twin boundaries.

To reveal the effect of *T*_s_ on the microstructure, SEM images of the three aforementioned films are shown in Figure 3. In Figure 3(a), the film grown at 400°C exhibits a continuous granular microstructure. If *T*_s_ = 600°C, as shown in Figure 3(b), the film surface is mostly uniform, with gaps and holes. In Figure 3(c), at *T*_s_ = 800°C, the film splits into isolated islands. For a more comprehensive observation, SEM images of eight films grown at 400–800°C are summarized in Figure S2 in the supplementary information. It clarifies that with lower *T*_s_ of 400 … 550°C, continuous granular microstructure forms. This is mainly because at low *T*_s_, the lack of thermal energy limits the diffusion ability of deposited atoms, which makes them unable to move freely on the substrate surface and thus many small nuclei form. These nuclei coalescent with their neighbors, resulting in the formation of a continuous film with a high density of grains and small-angle grain boundaries. In these cases, the films are still epitaxial. If *T*_s_ reaches 600°C and above, gaps and holes occur. At 700 and 800°C, grain islands are observed. These holes and islands mainly originate from the unfavorable high interface energy between film and substrate leading to dewetting [22,23]. The high interface energy probably originates from the large lattice mismatch. In Figure 3(c), as emphasized with a zoom-in image in the red box, parallel stripes are clearly visible, which is one more hint of an MT that the film has undergone during cooling from 800°C to room temperature.

### 3.3. Magnetic investigations

**Figure 4.**
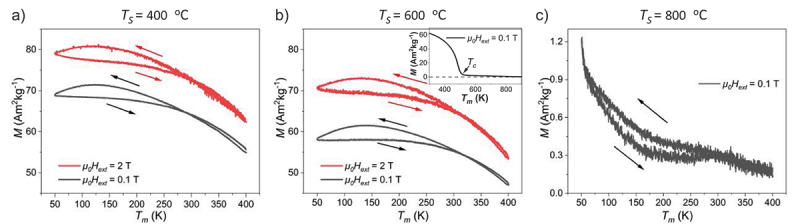
Temperature-dependent magnetization curves for three films deposited at a) 400°C, b) 600°C and c) 800°C reveal that with the increase of *T*_s_, the magnetization decreases. A hysteresis is observed in all cases between cooling and heating branches, which indicates for an increase of magnetocrystalline anisotropy at reduced temperature. *T*_c_ is measured at 515 K, as shown in the inset of b). Note a different magnetization scale in a, b) and c). The arrows illustrate the measuring direction.

**Figure 5.**
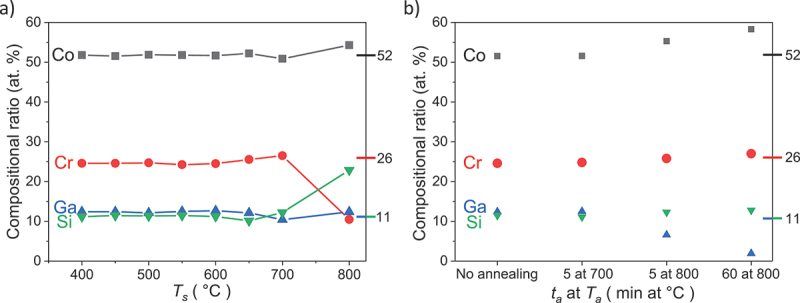
Summary of the elemental compositions of a) samples with varying *T*_s_ and b) samples modified by additional annealing. If either *T*_s_ or *T*_a_ exceeds 700°C, the composition differs from the target composition (marked at the right side of each graph).

In order to probe both martensitic transformation and magnetic transition, the magnetization as a function of the measurement temperature (*T*_m_) is studied, as summarized in Figure 4. We measure the magnetization in a lower (0.1 T) and a higher (2 T) external magnetic field, which is sufficient to reach saturation magnetization. For the film deposited at 400°C, as shown in Figure 4(a), the magnetization first increases during cooling. At *T*_m_ = 130 K, it reaches the maximum and decreases during further cooling. When heating, a hysteresis is observed which is not expected for a simple ferromagnetic behavior. To probe if the broad hysteresis originates from a MT, we measured temperature-dependent RSM at 300 K, 130 K and 90K in sequence (see Figure S3 in the supplementary). In all three RSMs, the austenitic peak (400)_A_ remains and no martensitic peaks appear. This allows excluding MT during cooling from 300 K to 90 K. Accordingly, our observation of a magnetic hysteresis indicates for a purely magnetic origin, e. g. an increased magnetocrystalline anisotropy within the ferromagnetic austenite at low temperatures. This anisotropic property becomes measurable in epitaxial films, in contrast to polycrystalline bulk. In addition, magnetization remains at a high level at the maximum *T*_m_ of 400 K, which suggests that *T*_c_ is higher than 400 K. In Figure 4(b), the film deposited at 600°C shows the same characteristic but with reduced magnetization. For probing *T*_c_, an additional measurement from 300 to 900 K with an oven set-up is performed, as shown in the inset of Figure 4(b). *T*_c_ is measured at 515 K. This film is in the mixed austenitic/martensitic state at room temperature and accordingly we expect that the martensitic ratio increases during further cooling. Indeed, the broad hysteresis in this *M*(*T*) curve opens just below room temperature. We attribute this to the martensite fraction in this sample, which commonly has a higher magnetocrystalline anisotropy due to the reduced crystal symmetry compared to the austenite. If *T*_s_ = 800°C, as shown in Figure 4(c), the magnetization is much lower than the other films. The *M*(*T*) result at 2 T (not shown) confirms that no ferromagnetic order is present in this film. For this film, we observe an anomaly steep increase of the magnetization below 250 K during cooling. This anomalous increase may originate from a RMT to austenite, as reported for bulk [7]. To directly probe the expected changes in crystal structure during cooling and heating, we measured temperature-dependent RSM at 300 K, 90 K, 500K, 600 K, 700 K and 775 K in sequence (see in Figure S4 in the supplementary) The martensitic peaks (224)_M_ and (004)_M_ are visible at all temperatures, but no austenitic peaks. Thus, neither a RMT occurs during cooling to 90 K, nor a MT during heating up to 775 K. Due to the limited temperature range accessible with this device, we could not probe a possible RMT in the temperature range between 90 and 50 K, where we observed the strongest increase of magnetization. The presence of martensite at the high temperature limit of 775 K indicates that the MT occurs somewhere above, just limited by the deposition temperature of 800°C (1073 K). Though with our available methods we cannot exclude that this films has been grown directly within the martensite state, we consider this unlikely. First, epitaxial growth within the martensite state should result in an artificial single variant state, as reported for the Fe_70_Pd_30_ system [24] and not observed here. Second, we measure two characteristic signatures of a MT: 1) twin boundaries (Figure 3(c)), and 2) the particular variant orientations expected by theory (Figure 2(c)). To sum up, for the influence of deposition temperature *T*_s_, we obtain the intended growth at all examined temperatures and films deposited below 650°C are ferromagnetic. However, as shown in the EDX data in Figure 5(a), if *T*_s_ reaches 800°C, the Cr content deviates from the initial level, which reveals the evaporation of Cr. In the following, we examine a different approach, which consists of deposition at moderate temperatures and subsequent annealing.

## 4. Tailoring film properties by additional annealing

Section 3 describes the influence of deposition temperature *T*_s_ on the structures and transformations behavior. These properties can also be controlled by a post-annealing process. Annealing can affect composition [25], release inner stress and change grain size. Annealing may also increase the degree of atomic order and thus enhance the saturation magnetization [26], shift the *T*_c_ and affect the transformation properties. It can also reduce the MT hysteresis and enhance martensitic stability. For that, we selected films grown at 450°C as the starting point to do the post-annealing, since this temperature is high enough for the desired epitaxial growth and low enough to avoid any evaporation. In the post-annealing process, the temperature *T*_a_ and the time *t*_a_ are the two controlled parameters. In section 3, we figured out that if *T*_s_ is over 600°C, the film starts to dewet. Moreover, if *T*_s_ reaches 800°C, the film splits into islands, and loses Cr as well as its ferromagnetism. Thus, for annealing, 700°C is a reasonable choice and 800°C is the limiting maximum. Therefore, the films are annealed for 5 min at 700°C and for 5 min and 60 min at 800°C.

### 4.1. Crystallographic orientations

**Figure 6.**
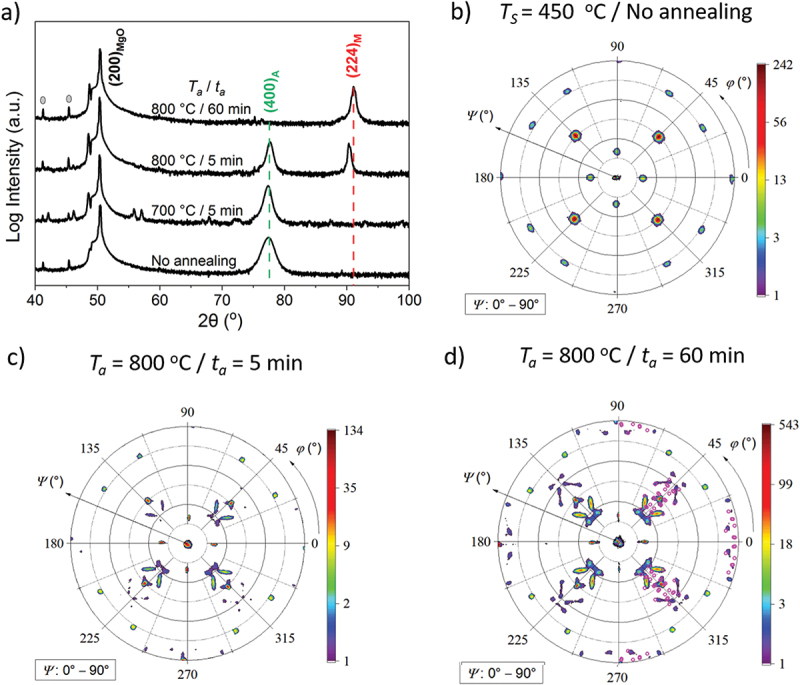
Influence of post-annealing on crystal structure, epitaxial growth and orientation relationships of the film deposited at 450°C, which is shown as a non-annealed reference: a), XRD θ/2θ scans of the annealing series show that with increasing annealing temperature and time, (400)_A_ reflection disappears and (220)_M_ appears. Peaks marked with grey dots are from the sample holder. b), the (220)_A_ pole figure of the film deposited at 450°C clarifies the epitaxial growth. c-d), the pole figures reveal that two films annealed at 800°C are partially martensitic at room temperature. In d), comparison of the calculated positions (pink in right half) using c/a = 1.258 [7] and the measured data (all other patterns) confirms that MT has occurred. Figure edges are parallel to the substrate edges.

To probe the effect of additional annealing on film crystallinity and epitaxy, as well as orientation relationships between the austenite and martensite phase, θ/2θ scans and pole figure measurements were performed on the three annealed films. Figure 6(a) shows that annealing at 700°C for 5 min still leaves the film in the austenite state at room temperature since only the (400)_A_ peak can be seen. After annealing at 800°C for 5 min, both (400)_A_ and (224)_M_ peaks are visible. A mix of martensite and austenite is obtained. The film annealed at 800°C for 60 min is completely martensitic since no austenitic peaks are observed. Figure 6(b–d) show the pole figures of the as-prepared film and the two films annealed at 800°C. The orientation relationship of the epitaxial film and the substrate was already discussed in section 3 (see Figure 2(a)). In Figure 6(b), four distinct peaks at *φ* = 45º, 135º, 225º and 315º come from the austenite. Figure 6(c) shows some features around the four main austenitic intensities, which matches well with the data in Figure 6(a), where diffraction peaks from both austenite and martensite are present. In Figure 6(d), the flower-like patterns confirm that during the martensitic transformation, the austenite splits up into many martensitic variants with different orientations. The measured positions match well with the pink patterns of the calculated values. To summarize, the film annealed at 800°C for 60 min is martensite with different variant orientations.

### 4.2. Microstructures

**Figure 7.**
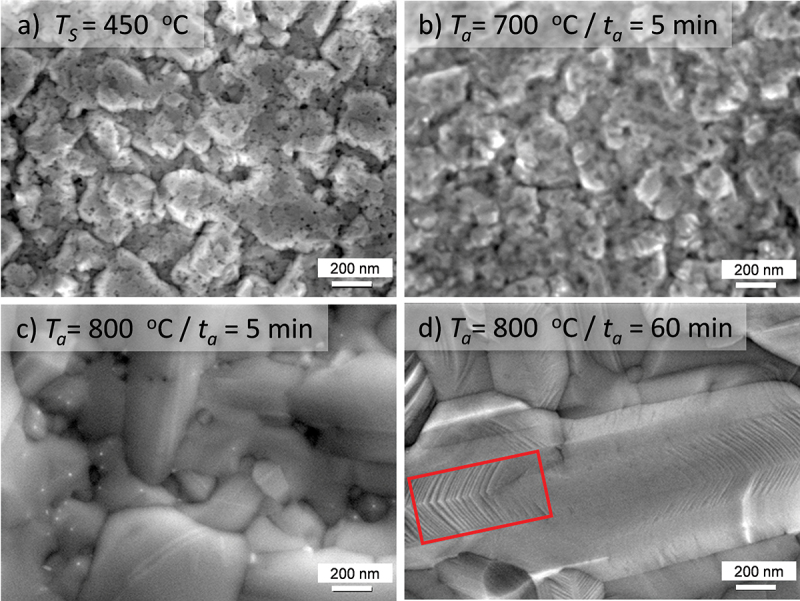
The microstructures change during post-annealing: from a-b) initial granular microstructure to c) large grains, and finally to d) martensitic microstructures with twin boundaries. The red box in d) marks the twin boundaries.

SEM in Figure 7(a) shows that, before annealing, the film exhibits a continuous morphology. After annealing at 700°C for 5 min (Figure 7(b)), no obvious change is observed, but the gaps between grains appear to be slightly reduced. After annealing at 800°C for 5 min (Figure 7(c)), we observe parallel stripes and lath-shaped microstructures. Moreover, the grain size increases and the size distribution becomes broader. If *T*_a_ stays at 800°C and *t*_a_ extends to 60 min (Figure 7(d)), the grain sizes increase further. The morphology still appears to be continuous in contrast to the island morphology of the film deposited directly at 800°C (Figure 3(c)). More interestingly, typical martensitic stripes and twin boundaries are clearly visible, as emphasized by a red box.

### 4.3. Magnetic investigations

To determine the influence of additional annealing on the magnetic properties, the specific magnetization *M* was measured as a function of the temperature *T*_m_ at a constant external field (*µ*_0_*H*_ext_ = 0.1 T). The measurement procedure is the same as used for Figure 4. The magnetization moment of the as-prepared film is 78.2 Am^2^kg^−1^ at *T*_m_ = 50 K, which suggests that the film is ferromagnetic. After annealing at 700°C for 5 min, *M* drops to 63.2 Am^2^kg^−1^. After 5 min at 800°C, only 4.5 Am^2^kg^−1^ is left, which is 5.8% of the initial value. Further, the magnetization is totally lost after heating at 800°C for 60 min. In all measurements, the cooling and heating curves practically overlap and accordingly there is no hint of any transformation occurring below 400 K. To sum up, for the influence of post-annealing, comparing to the section 3, after annealing at 800°C for 60 min, the film is martensitic and continuous with typical martensitic stripes and twin boundaries. However, the same as section 3, the film magnetization decreases drastically.

## 5. Origin of decreased ferromagnetic order

**Figure 8.**
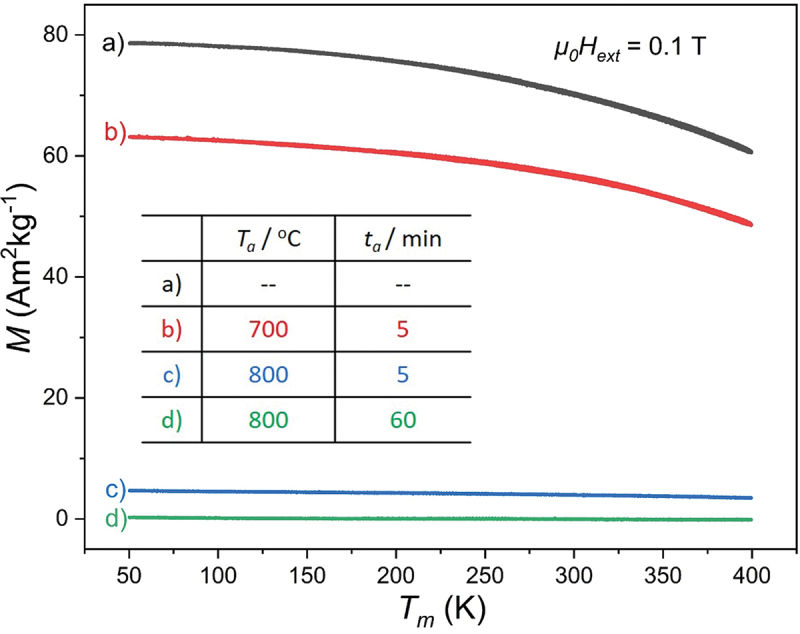
Influence of post-annealing on magnetization moment of a) the reference film deposited at 450°C and b-d) the annealed ones with the conditions shown in the inset table. The comparison shows that with increasing annealing temperature and time, the magnetization decreases. No obvious *M*(*T*) hysteresis is observed in any of the post-annealed samples.

In both the deposition and annealing cases, the *M*(*T*) curves show that the magnetization decreases strongly if the temperature reaches 800°C. We are still concerned about what changes in these films at 800°C, which might cause a sharp change of the magnetization and affect the MT. Since both of the two properties are linked with film composition, we first investigate the composition changes in dependence of *T*_s_ or annealing conditions by EDX. In Figure 5(a), if *T*_s_ ≤650°C, film compositions match well with the target value, as marked at the right side of each graph. If *T*_s_ exceeds 700°C, compositions no longer remain stable. At 800°C, it differs strongly from the target value because of evaporation of Cr. The loss of the strong ferromagnetic order (Figure 4(c)) might be attributed to the Cr evaporation. However, there is only half as much Cr as Co in the initial composition (a quarter after evaporation), but the magnetization moment drops by 99%. This huge impact cannot only be caused by the compositional shift towards less ferromagnetic elements. Assuming that the Co atoms contribute most of the magnetic moment, and the Cr atoms provide a strong coupling between the Co planes [27], the lattice distortion could also have a large impact on such coupling. Therefore, the more convincing reason for the loss of ferromagnetism is due to the formation of a non-magnetic martensite. This explanation matches the literature [7] that low magnetization was found to be a feature of the martensitic Co-Cr-Ga-Si. In the annealing case, however, regardless of the duration *t*_a_, after annealed at 800°C, film compositions change through the evaporation of Ga (Figure 5(b)) and the ferromagnetic order is lost (Figure 8(c,d)). The loss of ferromagnetism in this annealing case can also be attributed to the formation of a non-magnetic martensite during MT, which occurs in the partly martensitic film obtained at *T*_a_ = 800°C and *t*_a_ = 5 min and the completely martensitic film obtained at *T*_a_ = 800°C and *t*_a_ = 60 min. When comparing the composition changes, instead of using a higher deposition temperature, annealing at higher temperatures is not helpful for the compositional stabilization, since in the former case Cr is evaporated while in the latter case a decrease of Ga is observed. This indicates that two different mechanisms might be relevant, e.g. re-sputtering of Cr at a high deposition temperature and evaporation of Ga at a higher annealing temperature. As the latter becomes less relevant for thicker films, we propose to use thick films for future experiments with post-annealing.

## 6. Conclusions

In this work, we analyzed the conditions required to obtain epitaxial Co-Cr-Ga-Si films for analyzing their martensitic and magnetic transitions. We could show that epitaxial growth on MgO (100) is quite robust. Films are in ferromagnetic austenite phase if the deposition temperature is suitably low. Films grown at higher temperatures are partially martensitic at room temperature. Films at high deposition and annealing temperatures are fully martensitic and the ferromagnetism is almost lost. We attribute this loss to the MT from austenite to the non-magnetic martensite since low magnetization should be one of the features of martensitic Co-Cr-Ga-Si [7]. To obtain a martensitic film at room temperature by deposition or with annealing, high temperatures are required, while both results in the compositional changes. We propose to use thicker films and post-annealing to avoid this since our experiments reveal that the combination of moderate deposition temperatures followed by post-annealing allows the epitaxial growth and a continuous film morphology, which is beneficial for most experiments and applications. In one of these films, we observe a peculiarity in the magnetic behavior during cooling, which indicates a reentrant martensitic transformation. By temperature-dependent x-ray diffraction we can exclude this, which illustrates the need to combine independent methods to probe these multifunctional materials. In these experiments, the reentrant transformation is probably hindered by the low film thickness since the rigid substrate conditions often hinder transformation [28,29] and affect the magnetism [30]. Nevertheless, our work is the first to obtain epitaxial films as model systems to open up the exploration of RMT in thin films. Furthermore, we have strong indications, that within the Co-Cr-Ga-Si system MT above 775 K can be reached, which makes this system interesting as a high temperature shape memory alloy.

## Supplementary Material

Supplemental Material

## Data Availability

Experimental data is available at https://doi.org/10.14278/rodare.2123.
