# Supplementary material for: Growth and martensitic transformation of ferromagnetic Co-Cr-Ga-Si epitaxial films
Source: Sci Technol Adv Mater. 2023 Sep 11;24(1):2251368. doi: 10.1080/14686996.2023.2251368 (PMC10496528; doi:10.1080/14686996.2023.2251368)
Supplement: Supplemental Material [file TSTA_A_2251368_SM6514.docx]

**Supplementary materials: growth and martensitic transformation of ferromagnetic Co-Cr-Ga-Si epitaxial films**

Yuru Ge^1,2,5,6,^*, Klara Lünser^1,2,5^, Fabian Ganss^1^, Peter Gaal^3,4^, Lukas Fink^1,2,5^ and Sebastian Fähler^1,2^

^1^Helmholtz-Zentrum Dresden-Rossendorf, Institute of Ion Beam Physics and Materials Research, 01328 Dresden, Germany

^2^Leibniz IFW Dresden, Institute for Metallic Materials, 01069 Dresden, Germany

^3^Leibniz-Institut für Kristallzüchtung (IKZ), 12489 Berlin, Germany

^4^TXproducts UG, Luruper Hauptstraße 1, 22547 Hamburg, Germany

^5^TU Dresden, Faculty of Mechanical Science and Engineering, 01062 Dresden, Germany

^6^TU Chemnitz, Faculty of Natural Sciences, 09107 Chemnitz, Germany


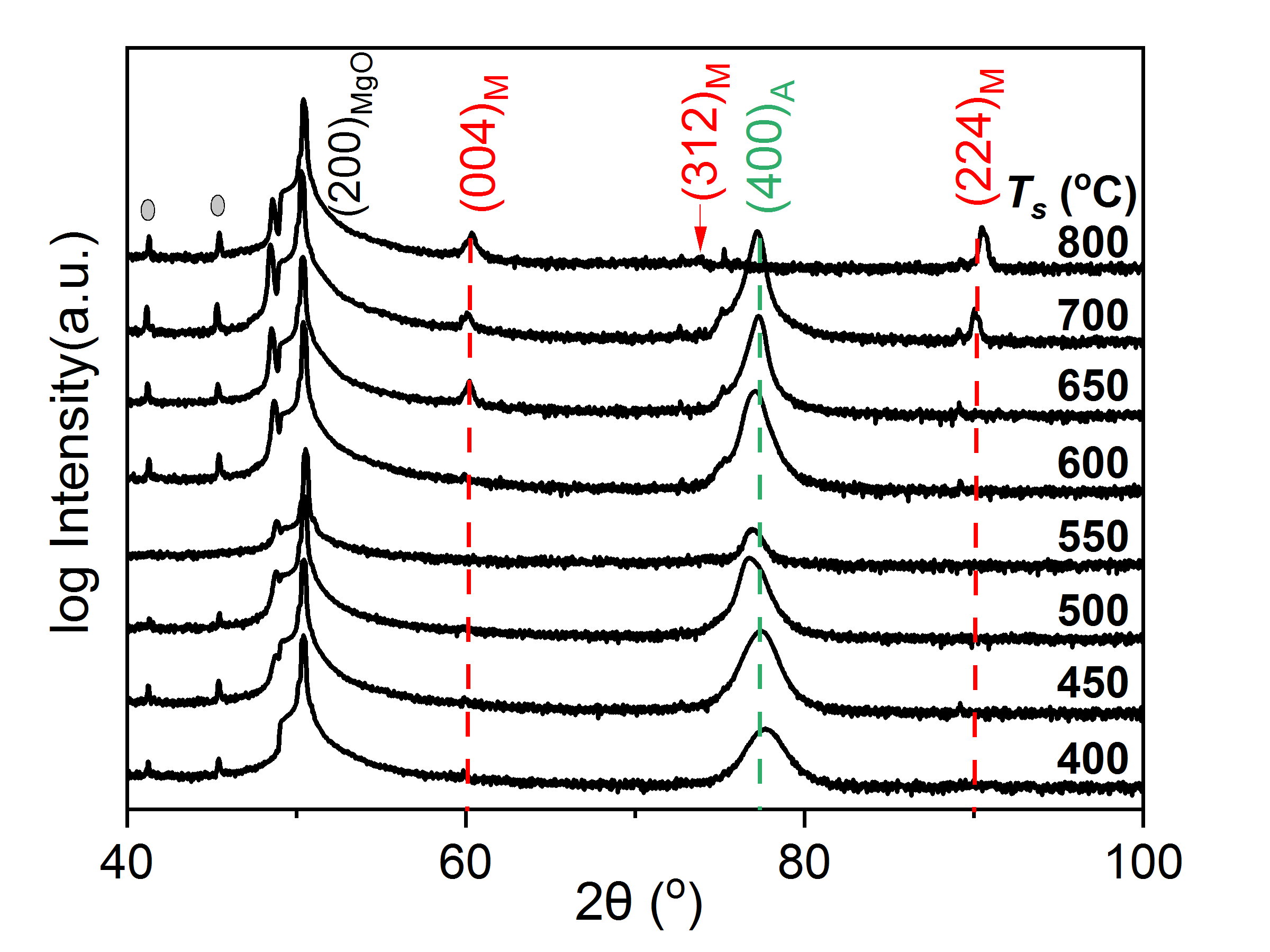


Figure s1 XRD θ/2θ scans with χ = 0º of Co-Cr-Ga-Si films at different deposition temperatures *T*_s_. Diffractions are donated by (400)_A_ which gives the first hint that films grow epitaxial or at least with one dominant orientation. (004)_M_, (312)_M_ and (224)_M_ are clear seen when *T*_s_ reaches 700 ºC. This means that films grown below 650 ºC are mainly austenitic at room temperature, while films deposited at 650 ºC and above are more and more martensitic. Reflections marked with grey circles come from the set-up.

Fig. s1 shows the XRD standard Bragg-Brentano measurements with θ/2θ scans at χ = 0º of the involved 8 Co-Cr-Ga-Si films at different deposition temperatures *T*_s_, in contrast to Fig. 1a, which are the sum for χ tilts from 0º to 10º. Accordingly, here only reflections from lattice planes, which are strictly parallel to the substrate occur, but no reflections of tilted martensitic variants are visible. If the deposition temperature *T*_s_ < 600 ºC, the (400)_A_ reflection is observed and no peaks from other orientations. It confirms that the films grown below 600 ºC is mainly austenitic. If 600 ºC ≤ *T*_s_ ≤ 700 ºC, austenitic peak (400)_A_ and martensitic peaks (004)_M_ and (224)_M_ are observed, which means these films are a mixture of austenite and martensite. If *T*_s_ = 800 ºC, only three martensitic peaks (004)_M_, (312)_M_ and (224)_M_ are observed, proving that this film deposited at 800 ºC is martensitic. Thus, the transformation will only happen when films deposited at 600 ºC or higher are cooled down after deposition.


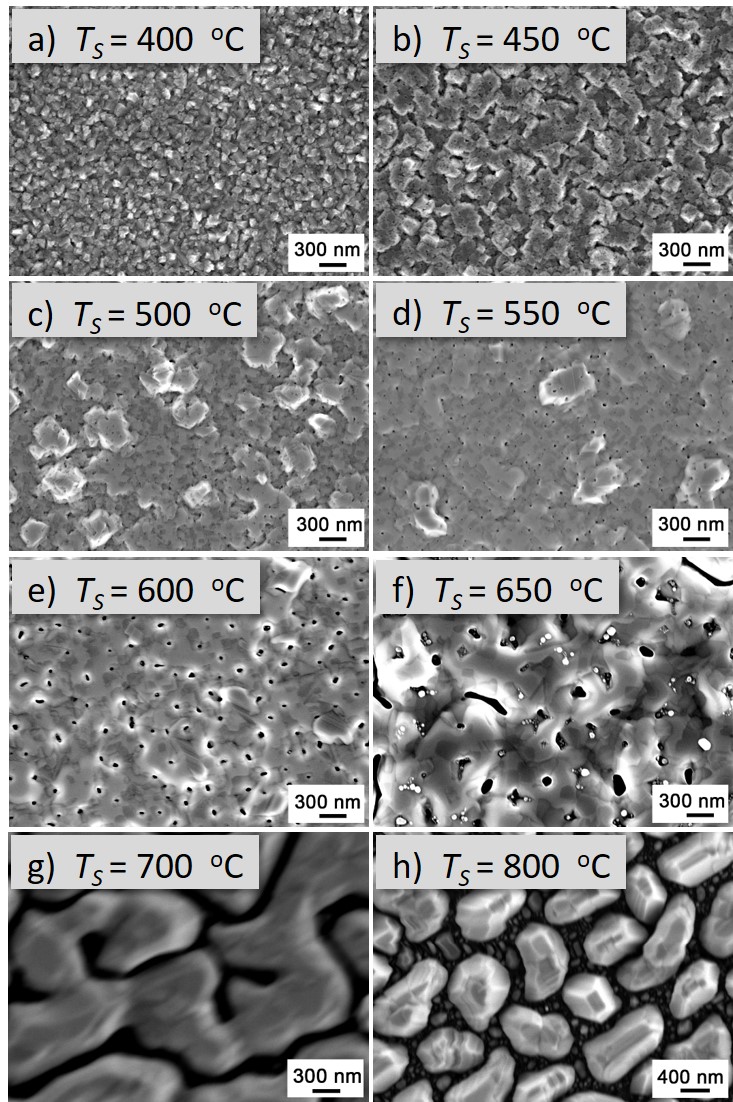


Figure s2 The microstructure of the films changes with the deposition temperature *T*_s_: SEM images of the films deposited on MgO (100) with the *T*_s_ of a), 400, b), 450, c), 500, d), 550, e), 600, f), 650, g), 700 and h), 800 ºC, respectively.

**Temperature-dependent reciprocal space mapping measurements**

To elucidate the structural changes within the austenitic film deposited at 400 ºC and the martensitic film deposited at 800 ºC, x-ray reciprocal space mappings (RSM) were recorded from 90 K up to 775 K. The measurements were done with a Rigaku SmartLab diffractometer with a 1D detector and Cu Kα-radiation. We use an Anton Paar DHS 1100 heating stage to reach the measurements under ambient conditions in vacuum. Cooling to 90 K is achieved by a liquid N_2_ set-up. The results are shown in Fig. s3 and Fig. s4

For the film deposited at 400 ºC, we started with a RSM measurement at 300 K, as shown in Fig. s3(a). Then, we cooled the film to 130 K which is at the max. hysteresis in Fig. 4a and recorded the next RSM, as shown in Fig. s3(b). Then we cooled the film to 90 K, which is the minimum temperature we can reach with liquid N_2_ cooling. A RSM was recorded after temperature stabilized (Fig. s3(c)).

For the film deposited at 800 ºC, we measured a RSM at 300 K, as shown in Fig. s4(a). Then, the film was cooled to 90 K which is the min. temperature we can reach and a RSM was recorded (Fig. s4(b)). Then we heated the film to 500 K, 600 K, 700 K and 750 K in sequence and recorded a RSM in each step when the temperature was steady, as shown in Fig. s4(c-f).

The rings visible in all RSMs come from the polycrystalline Cu sample holder since the beam size is larger than the sample which we had to be cut for previous VSM measurements.


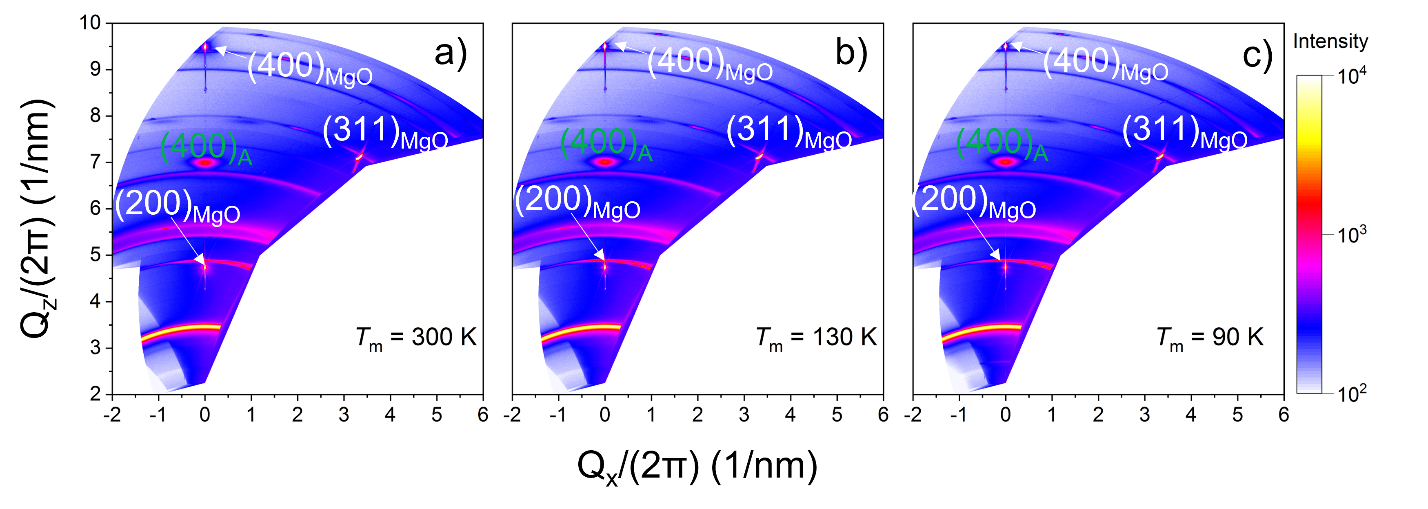


Figure s3 Temperature-dependent RSM measurements of the film deposited at 400 ºC at (a) 300 K, (b) 130 K and (c) 90 K show that there is no martensitic transformation during cooling to 90 K. Accordingly, our observation of a magnetic hysteresis in figure 4(a) indicates for a purely magnetic origin, e. g. an increased magnetocrystalline anisotropy in austenite at low temperatures. This result matches the magnetic measurement in figure 4(a), where the film is still ferromagnetic when cooled to 50 K, which is a feature of the Co-Cr-Ga-Si austenite.


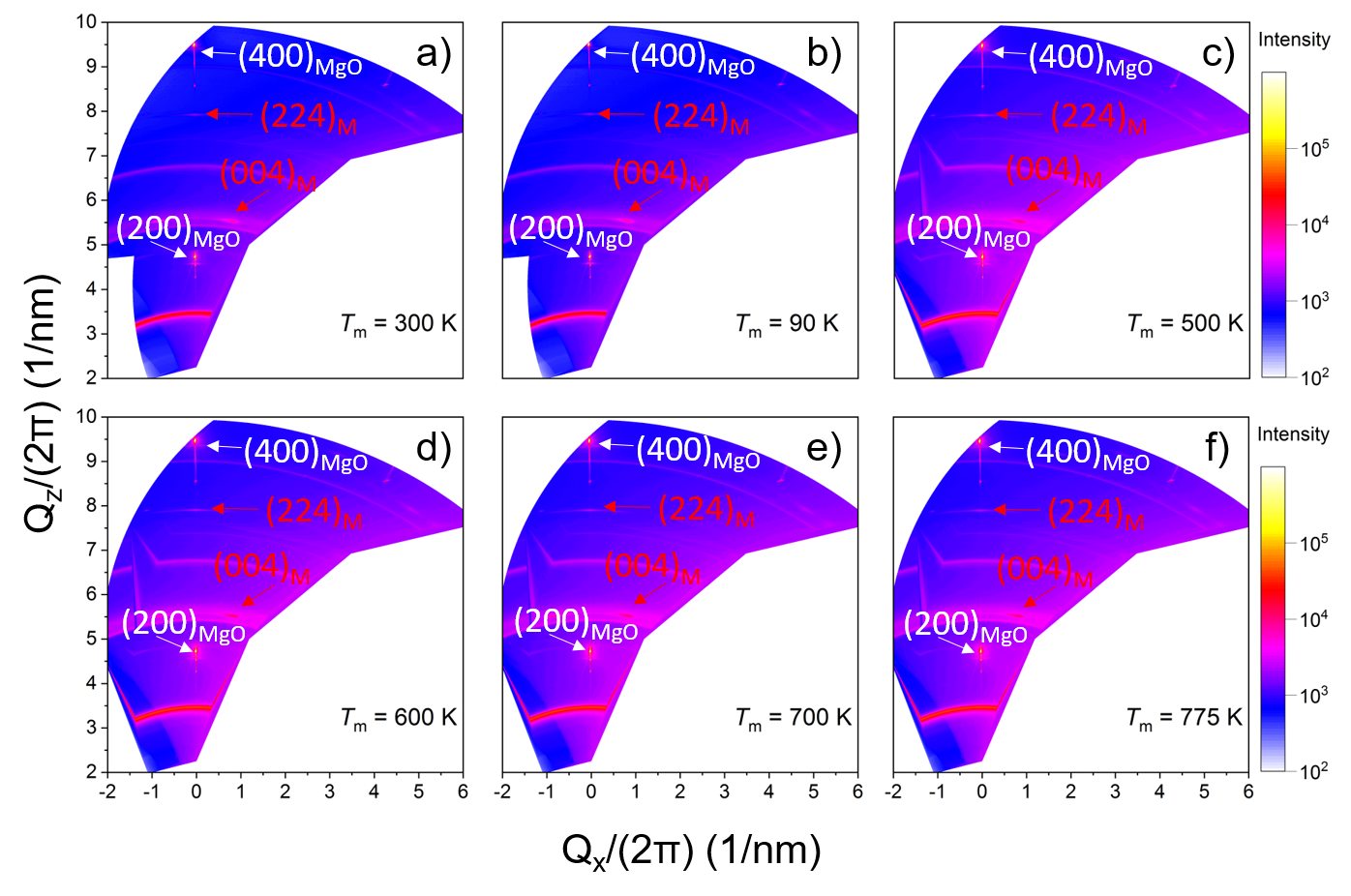


Figure s4 Temperature-dependent RSM measurements of the film deposited at 800 ºC at (a) 300 K, (b) 90 K, (c) 500 K, (d) 600 K, (e) 700 K and (f) 775 K show that there is no RMT during cooling to 90 K and no conventional transformation during heating to 775 K.
